# Editorial: Tobacco industry accountability - Current practices, emerging issues and challenges

**DOI:** 10.3389/fpubh.2023.1220268

**Published:** 2023-08-10

**Authors:** Sonu Goel, Amit Yadav, Anna Kontsevaya, Garima Bhatt

**Affiliations:** ^1^Department of Community Medicine, School of Public Health, Post Graduate Institute of Medical Education and Research (PGIMER), Chandigarh, India; ^2^Public Health Master's Program, School of Medicine and Health Research Institute (HRI), University of Limerick, Limerick, Ireland; ^3^Faculty of Human and Health Sciences, Swansea University, Swansea, United Kingdom; ^4^Department of Tobacco and NCD Control, International Union Against Tuberculosis and Lung Disease (The Union), South-East Asia Office, New Delhi, India; ^5^National Research Center for Preventive Medicine, Moscow, Russia

**Keywords:** tobacco industry (TI), interference, accountability, challenges, FCTC, Article 5.3

## Tobacco industry accountability

The long and intricate history of the tobacco industry (hereafter TI) is marked by periods of expansion and disintegration, changing legal frameworks, and evolving public perception of tobacco use and its detrimental effects on health (1).

The global TI is dominated by the five largest tobacco businesses, i.e., Philip Morris International, British American Tobacco, Imperial Brands, Japan Tobacco International, and China National Tobacco Company (2). These are also known to historically work together in concealing scientific evidence on the adverse effects of tobacco consumption, telling lies under oath before the US Congress, and manipulating and destroying evidence (3).

Since the mid-20th century, the evidence against tobacco products has been mounting, linking tobacco use (smoked as well as smokeless forms) to various cancers and other health problems (4). There is evidence that smoking leads to many cancers, not just lung cancer (5).

In response, tobacco companies launched aggressive marketing campaigns to reassure the general population about the safety of their products and undermine the credibility of scientific research on the harms of smoking (1). In the 1990's, public pressure and lawsuits led to a series of major legal settlements and the implementation of tighter regulations on tobacco advertising and sales (6). To circumvent this new regulatory environment, numerous tobacco firms expanded their product portfolios to include smokeless tobacco and other items containing nicotine (7). More recently, the industry has also moved its focus onto creating and marketing substitutes, notably, heated tobacco products and electronic cigarettes, misleading consumers with the term “harm reduction,” or “less harmful,” or “safer” used for such products (8, 9). While TI has long been criticized and subject to legal action due to its part in promoting and making money from a harmful and addictive product, TI has employed several tactics to counter the legal and public health regulations put in its way by governments in different countries (9).

## Tobacco industry tactics

It is well-documented that the TI has been using various tactics to interfere with public health policies and programs that reduce tobacco use and its associated harms (10). TI has been funding research studies that discredit proven science by sponsoring and promoting research that produces results biased in favor of its products and using them to influence public health policy through lobbying and other forms of political influence (11, 12). TI has also used its financial resources and political influence to lobby against public health policies, including tobacco taxes, smoke-free laws, advertising restrictions, and facilitating illicit trade in tobacco products through smuggling (12–15). This has included funding political campaigns and candidates sympathetic to the industry's interests and sponsoring front groups and other organizations that advocate for the industry's position (16). In addition, the TI continues to introduce and market newer products (e-cigarettes and heated tobacco products) and create a misleading perception of being a healthier option compared to traditional tobacco products through social media influencers and product placements in movies and television shows (17). Furthermore, TI has also twisted and exploited trade and other international agreements to undermine public health policies (18). Additionally, TI has used front groups to aggressively lobby for pro-industry measures influence the political and legislative process, promote misinformation, and exaggerate the industry's economic importance (19).

However, since the adoption of the World Health Organization- Framework Convention on Tobacco Control (WHO-FCTC), there have been demands for the industry to be held to a higher degree of accountability and to firewall tobacco control policies from deceitful and deceptive interference by the industry. The WHO-FCTC is a global evidence-based treaty that was developed in response to the tobacco epidemic's globalization that asserts everyone's right to the highest standard of health (20).

## WHO-FCTC Article 5.3

Article 5.3 of the FCTC and the guidelines adopted for its effective implementation recommend that TI and those working to advance its interests operate and act in an accountable and transparent manner (21). These guidelines are intended to ensure tobacco control measures are thorough and successful in averting commercial and other ingrained interests of the TI. These guidelines and principles cover interferences by TI and, as apposite (as appropriate), by individuals and organizations that work to advance the interests of the TI further (21).

Unfortunately, as more victims of tobacco use epidemic have increased, the TI sees itself as part of the solution and not the problem. It uses various tactics to stymie the government's effort to reduce tobacco users and protect public health. However, the key to tackling Tobacco Industry Interference (henceforth TII) lies in the hands of governments that adopt a comprehensive policy against TII that aligns with the WHO FCTC. Article 5.3 of the WHO FCTC requires that:

“*In setting and implementing their public health policies with respect to tobacco control, Parties shall act to protect these policies from commercial and other vested interests of the tobacco industry in accordance with national law.”*

## Against all odds

Australia became the first nation to implement plain packaging regulations on tobacco products, limiting cigarette packs' branding and other promotional features in 2012. The TI contested the law in court, arbitration tribunal, and World Trade Organization (WTO), but the Australian government finally prevailed, which is believed to have reduced smoking rates nationwide (22).

Uruguay introduced several policies in 2010 to lower tobacco consumption, including banning smoking in public areas and prohibiting tobacco advertising and promotion. The tobacco industry challenged these measures, but the measures implemented by Uruguay prevailed (23).

Thailand's Ministry of Public Health filed a successful lawsuit in 2017 against the Thai subsidiary of Philip Morris International, alleging that the company had imported and sold cigarettes that did not comply with the country's regulations (24).

India notified rules requiring 85% pictorial health warnings on the packaging of tobacco products in 2014. The TI pushed back against the move. The industry filed legal challenges, claiming that the warnings were too graphic, and even closed down manufacturing units in an attempt to get their way by disrupting the economy of India. However, TI lost with landmark ruling by the Supreme Court of India that upheld the implementation of larger and stronger health warnings on all tobacco products in India (25).

The current theme of this Research Topic has meticulously captured the challenges TI poses in undermining public health practices and opportunities for intervention, along with key takeaways for policymakers to implement stronger actions. The issue comprises ten articles, two of which focus on smokeless tobacco, highlighting the degree of surrogate advertisement of Smokeless Tobacco (SLT) products and the development and assessment of a Stop Spit Tobacco Curriculum. Two manuscripts present secondary data analysis, focussing on TI's influence on tobacco use among young people and the use of multiple imputation methods to handle missing values in panel data. Three articles shed light on harm reduction and commercialized harm reduction, examining influencer-vaping brand relationships on Instagram for compliance with advertising regulations and analysis of social media marketing of e-cigarettes in countries with different regulatory policies. In addition, three papers discuss challenges in monitoring diplomats' engagement with the TI, using price-policy measures, and implementing tobacco cessation strategies to manage tobacco-induced disease burden.

The issue covers articles that bring forth several maneuvers of the TI in the current times, including the digital and social media marketing tactics for advertising Electronic Nicotine Delivery Systems (ENDS)/non-combustibles and building the narrative of harm reduction, flouting advertising regulations—brand stretching and surrogate advertising, misusing the principles of human rights to advance commercial interests of harm reduction, marketing new dissolvable tobacco products, the actions of diplomats, which contravene the tenets and guidelines of WHO-FCTC, and attempts by TI to access laws, retailers' opposition, and suboptimal enforcement and access to cigarettes at unregulated alternative vends (outlets).

## Way forward

In order to counter these evolving challenges, it is crucial to promote evidence-based public health policies, transparency, and accountability for the tobacco industry's actions. It is essential for governments to continuously monitor all forms of media, including digital and social media, to track the online promotion and advertising of new tobacco/nicotine products and to strengthen counter-response for the same. Furthermore, the countries Party to or who have ratified WHO-FCTC should ensure that government representatives abide by provisions of Article 5.3 at both national and international levels. There is a need for strict regulation of TI behavior with effective enforcement of laws prohibiting tobacco advertising, including Corporate Social Responsibility (CSR) and other corporate promotions by the TI. Finally, building a firewalled collaboration and partnership across public health advocates, civil society organizations, and government agencies, as well as engaging academic researchers, legal experts, the public, and the media can help to highlight the industry's tactics, support evidence-based policies to advance tobacco control and prevent such policies from any undue TII.

## Author contributions

SG, AY, and AK conceptualized the idea. GB undertook the review of the literature and prepared the first draft. SG and AY reviewed the draft and gave technical input to the original draft. All authors contributed to the article and approved the submitted version.
